# Role of Genetic Polymorphisms -238 G>A and -308 G>A, and Serum TNF-α Levels in a Cohort of Mexican Pediatric Neuroblastoma Patients: Preliminary Study

**DOI:** 10.3390/ijms251910590

**Published:** 2024-10-01

**Authors:** Arturo Ramírez-Pacheco, Silvia Selene Moreno-Guerrero, Luz María Rocha-Ramírez, Gabriela Hernández-Pliego, María Argelia Escobar-Sánchez, Alfonso Reyes-López, Juan José Luis Sienra-Monge, Luis Enrique Juárez-Villegas

**Affiliations:** 1 Departamento de Hemato-Oncología, Hospital Infantil de México Federico Gómez, Dr. Márquez No. 162, Col Doctores, Ciudad de México 06720, Mexico; artur_tauro@yahoo.com.mx (A.R.-P.); sswitch@yahoo.com (S.S.M.-G.); aleirbagy@hotmail.com (G.H.-P.); 2 Unidad de Investigación en Enfermedades Infecciosas, Hospital Infantil de México Federico Gómez, Dr. Márquez No. 162, Col Doctores, Ciudad de México 06720, Mexico; 3 Departamento de Patología Clínica y Experimental, Hospital Infantil de México Federico Gómez, Dr. Márquez No. 162, Col Doctores, Ciudad de México 06720, Mexico; bcnhim2009@hotmail.com; 4 Centro de Estudios Económicos y Sociales en Salud, Hospital Infantil de México Federico Gómez, Dr. Márquez No. 162, Col Doctores, Ciudad de México 06720, Mexico; alfonso.reyes.lopez@outlook.com; 5 Subdirección de Pediatría Ambulatoria, Hospital Infantil de México Federico Gómez, Dr. Márquez No. 162, Col Doctores, Ciudad de México 06720, Mexico; jjsienra@hotmail.com

**Keywords:** neuroblastoma, TNF-α, polymorphisms, cancer susceptibility, survival, prognosis

## Abstract

The results of in vitro and in vivo studies have shown the pro-tumor effects of TNF-α, and this cytokine’s increased expression is associated with poor prognosis in patients with some types of cancer. Our study objective was to evaluate the possible association of TNF-α genetic polymorphisms and serum levels with susceptibility and prognosis in a cohort of Mexican patients with NB. We performed PCR-RFLP and ELISA methods to analyze the genetics of these SNPs and determine serum concentrations, respectively. The distribution of the -308 G>A and -238 G>A polymorphisms *TNFα* genotypes was considerably different between patients with NB and the control group. The SNP rs1800629 GG/GA genotypes were associated with a decreased risk of NB (OR = 0.1, 95% CI = 0.03–0.393, *p* = 0.001) compared with the AA genotype, which was associated with susceptibility to NB (OR = 2.89, 95% CI = 1.45–5.76, *p* = 0.003) and related to unfavorable histology and high-risk NB. The rs361525 polymorphism GG genotype was associated with a lower risk of developing NB compared with the GA and AA genotypes (OR = 0.2, 95% CI = 0.068–0.63, *p* = 0.006). Circulating TNF-α serum concentrations were significantly different (*p* < 0.001) between patients with NB and healthy controls; however, we found no relationship between the analyzed TNF-α serum levels and SNP genotypes. We found associations between the rs1800629AA genotype and lower event-free survival (*p* = 0.026); SNP rs361525 and TNF-α levels were not associated with survival in patients with NB. Our results suggest the TNF-α SNP rs1800629 as a probable factor of NB susceptibility. The -308 G/A polymorphism AA genotype has a probable role in promoting NB development and poor prognosis associated with unfavorable histology, high-risk tumors, and lower EFS in Mexican patients with NB. It should be noted that it is important to conduct research on a larger scale, through inter-institutional studies, to further evaluate the contribution of *TNF-α* genetic polymorphisms to the risk and prognosis of NB.

## 1. Introduction

Neuroblastic tumors (NB) are the third most common solid tumors in children, accounting for about 10% of malignant neoplasms in pediatric patients and representing 10–15% of mortality from pediatric tumors [1,2]. In Mexico, its incidence is around 2.9–3.6% per one million children/year [3,4,5]. Most patients are diagnosed in the advanced stages of the disease, decreasing disease-free survival [3,4,6,7]. In addition, metastasis is another factor related to poor clinical results, with bone metastasis occurring most frequently [3,4,6,7,8]. This tumor presents considerable histopathological, morphological, biological, genetic, and clinical heterogeneity, which can decisively influence prognosis [3,8,9,10].

Cytokines produced by cell interactions in the tumor microenvironment play an important role in the pathogenesis of cancer [11]. Non-malignant cells in the tumor microenvironment can positively or negatively affect the growth, survival, and metastatic potential of tumor cells, including NB [12,13]. Alternatively, malignant cells may respond to host-derived cytokines that promote growth, attenuate apoptosis, and facilitate invasion and metastasis [11]. Tumor necrosis factor α (TNF-α) is a pro-inflammatory cytokine produced by both malignant and immune cells within the tumor-associated microenvironment and contributes to the development and progression of malignant disease by creating an inflammatory niche that supports the tumor [12]. TNF-α is a powerful immunomodulator implicated in growth factor and cytokine activation, such as in IL-8 and MCP-1, which display chemo-attractant activity that affects the synthesis and alteration of adhesion molecules. Furthermore, TNF-α promotes inflammatory response and is critical in the pathogenesis of inflammatory, autoimmune, and malignant diseases [14]. TNF-α displays pro-tumor activity by stimulating the growth, proliferation, invasion, angiogenesis, and metastasis of cancer cells by activating nuclear factor kB (NF-kB), an important cell survival signal that inhibits the apoptotic process [12,15]. On the other hand, TNF-α suppresses tumor growth through its anti-proliferative, cytostatic, and cytolytic effects against various malignant cells; however, many of these cells are resistant to TNF-α-induced cytotoxicity [16]. TNF-α acts through its two distinct receptors: TNFR1 (TNF receptor 1) is expressed on all cells in the body, and TNFR2 (TNF receptor 2) is expressed primarily on immune suppressor and neoplastic cells. TNFR1 has an important role in apoptotic cell death and TNFR2 seems to have a tumor-promoting effect [17]. Both receptors transduce their signals cooperatively [18]. Recently, the interaction between TNFR2 of the tumor cell and the TNF-α bound to the monocyte membrane was reported, triggering tumorigenic inflammation in NB [19].

The TNF-α coding gene is located on chromosome 6p21.3. It is 3 kb in size; contains four exons; has several single nucleotide polymorphisms (SNPs), two of which are in the gene’s promoter region; and can affect the production of TNF-α [20]. The -308 G/A (rs1800629) and -238 G/A (rs361525) SNPs are found in a region close to the transcription site, resulting in differences in gene expression and TNF-α protein secretion [21]. The -308 G/A polymorphism is related to an increase in *TNF-α* gene transcription supported by gene reporter assays [14,21]. Substituting a guanine (G) base for adenine (A) in position 308 generates two allelic forms: the wild-type -308G and the variant-type -308A. The presence of the -308A allele correlates with increased production of TNF-α in vitro and in vivo [21,22]. It has been documented that transcriptional activity increases severalfold and secretion of this cytokine increases when the A allele is present [21,22]. The GA and AA genotypes have been associated with higher mRNA levels and elevated serum TNF-α concentrations compared with individuals with the GG genotype [23,24,25]. The -308A allele is linked to clinical susceptibility to various diseases. In vitro research results reported its links to increased TNF-α production [26]. However, SNP -238 G/A is produced from substituting a guanine (G) base for adenine (A) in position 238, generating the wild- and variant-type alleles, -238G and -238A. This SNP is associated with reductions in *TNF-α* gene transcription [27]. The G allele of the -238 polymorphism has been shown to be associated with elevated TNF-α production compared with the A allele [28]. However, reports are limited, and they still do not provide clear evidence of this SNP’s role in regulating *TNF-α* gene transcription. The -238 A allele is a protective factor in disease susceptibility [27,29,30], and based on reports from meta-analyses, both polymorphisms are associated with susceptibility and clinical outcomes in various inflammatory diseases, infections, and cancer [21,26,27,28,29]. In addition, bioinformatic analyses of online databases describe positive associations of SNPs in genes encoding various cytokines with several human diseases, including the polymorphisms -238 G/A (rs361525) and -308 G/A (rs1800629) of *TNF-α* [31].

It has been reported that the distribution of polymorphisms in genes of several cytokines such as TNF-α distinguishes the Mexican population from other groups, including Caucasians, Asians, and Africans, showing a different distribution for the AG haplotype, made up of the polymorphisms -238 G/A and -308 G/A of *TNF-α*, in the Mexican population (7.2%), Asian population including Chinese (8.8%) and Koreans (8.2%), and two European populations Romanians (13.2%) and Macedonians (14%) [32]. Likewise, published data on frequencies of the A allele of SNP rs1800629 shows differences between European (5–10%), African (0.8–14%), South African (5%), British White Caucasian (30%), Asian (0–2%), and Mexican (0–8%) populations [14,33,34].

The A allele for the rs361525 variant in heterozygous genotype has been reported to be present with a frequency of around 0.1–0.2% in different populations [35,36]. Since these *TNF-α* polymorphisms are associated with the development of various diseases, knowledge of the distribution of these SNPs in the Mexican population may be useful to understand their role as possible genetic factors of susceptibility or prognosis in our population.

*TNFα* genetic polymorphisms can influence the levels of this cytokine in vivo in various conditions. The modulation of immune responses, as in TNF-α-dependent cell death processes, has long been the subject of intense investigation. However, its role in cancer progression is complex and controversial because of this cytokine’s involvement in cancer development, which may have opposite effects on tumor growth [37,38,39].

There are no reported studies on the implications of circulating concentrations and TNF-α genetic polymorphisms on NB. Therefore, the purpose of our study was to evaluate the association of the -238 G/A rs361525 and -308 G/A rs1800629 genetic polymorphisms and serum levels of TNF-α with the clinical pathological factors and disease susceptibility in a cohort of children with NB.

## 2. Results

Our study population was 27 pediatric patients with NB, the majority (59.3%) were aged <18 months at diagnosis, and 37.03% were men and 62.9% were women. More than half of the subjects were classified as high risk (55.6%), and 48.1% were at INSS stage 4 (Table 1). The most frequent primary tumor location was in the retroperitoneal space (40.7%), and the majority presented partially differentiated tumors (59.3%) (Table 1). A total of 55.5% presented metastases at diagnosis, only 3.7% had amplification of the *MYCN* gene, and 25.9% of the patients relapsed, with 22.2% dying during the follow-up. In the control group of 38 healthy pediatric subjects, who were matched with the NB cases by age and sex, none presented amplification of the *MYCN* gene (Table 1).

### 2.1. Distribution of TNF-α Genetic Polymorphisms between NB Cases and Control Group

The genotype and allele frequencies presented important differences between cases and controls for the analyzed SNPs (Table 2). For the SNP rs361525, the homozygous wild-type GG genotype occurred more frequently in healthy controls compared with NB cases (81.6% vs. 48.1%); on the contrary, the heterozygous GA and homozygous variable AA genotypes occurred more frequently in NB cases in relation to controls (18.4% vs. 40.7%) and (0% vs. 11.1%), respectively (Table 2). Furthermore, the AA genotype was not found in the analyzed healthy controls. For SNP rs1800629, the homozygous wild-type GG was the most frequent in controls and cases with NB but with a marked difference in percentage, being much higher for controls compared with cases (86.8% vs. 48.2%). We observed that the heterozygous GA (2.6% vs. 14.8%) and homozygous wild-type AA (10.5% vs. 37%) genotypes were more frequent in patients with NB compared with the control group. Our logistic regression analysis revealed that the rs361525 polymorphism GA genotype was associated with (*p* = 0.004) increased risk of presenting NB (OR 4.52; 95% C.I. 1.61–12.6) compared with the wild-type homozygous GG genotype (Table 2). For the rs1800629 variant, the AA genotype may increase the risk of disease compared with the homozygous wild-type GG genotype (*p* = 0.003) (OR 2.89; 95% C.I. 1.452–5.765) (Table 2).

### 2.2. Analysis of NB Patient Genotypes and Prognostic Factors

We observed that most of the patients who presented high-risk prognostic factors had at least one G allele for rs361525 *TNF-α* polymorphism; however, our analysis did not show a significant association with any of the analyzed prognostic factors (Table A1). For the rs1800629 *TNF-α* polymorphism, the GA + AA genotypes showed a significant association (*p* = 0.038) with the presence of unfavorable histology and were at the limit of significance (*p* = 0.05) with high-risk category and INSS stage 4; however, there were no associations with other prognostic factors (Table A1).

A simple logistic regression analysis was performed to evaluate the association between *TNF-α* genetic polymorphisms and prognostic factors. For SNP rs361525, we found that carriers of the GG and GA genotypes have intermediate/high-risk NB (OR 1.5, 95% CI 0.20–10.8); however, this association is at the limit of significance (*p* = 0.05). In addition, the AA genotype was found to be significantly associated (*p* = 0.017) with a decreased risk of developing neuroblastic tumors with INSS stages 3 and 4 (OR 0.043, 95% CI 0.003–0.57) (Table 3). The remaining prognostic factors we evaluated did not present an important relationship with this SNP (Table 3).

For SNP rs1800629, a high frequency of patients with the GG genotype had favorable histology; however, this association was not significant. On the contrary, the patients with the GA/AA genotype were more likely to display high-risk NB (*p* = 0.04) and unfavorable histology (*p* = 0.03) (Table 4). With these findings, we can suggest that the A allele of this polymorphism may probably be a genetic biomarker of poor prognosis in Mexican patients, associated with unfavorable advanced-stage neuroblastic tumors.

### 2.3. Relationship of TNF-α Serum Levels Between Cases of NB and Control Group

Our analysis of TNF-α serum levels between the NB patients (n = 27) and control group (n = 27) showed significant differences (*p* < 0.001), indicating that the serum concentrations of this cytokine may probably have implications in the development of NB, due to the pro-tumoral role of TNF that has been reported in previous studies (Table 5). However, we considered low ≤ 20 pg/mL and high levels ≥ 21 pg/mL in the stratification of TNF-α serum levels. Based on this, 88.9% of patients with NB had low levels of TNF-α. All healthy pediatric subjects showed low levels of TNF-α (Table 5).

### 2.4. Association of Genotypes and Serum Levels

Our evaluation of the distribution of the genotypes rs1800629 and rs361525 of TNF-α with respect to serum levels in NB patients did not reveal a significant association of any genotype for both polymorphisms studied with circulating levels of TNF-α (Table A2). We observed that more than 40% of NB patients with homozygous wild-type (GG) genotypes for these polymorphisms presented low TNF-α serum levels compared with patients with heterozygous and homozygous-variant genotypes (Table A2).

### 2.5. Survival Analysis

**Figure 1:**
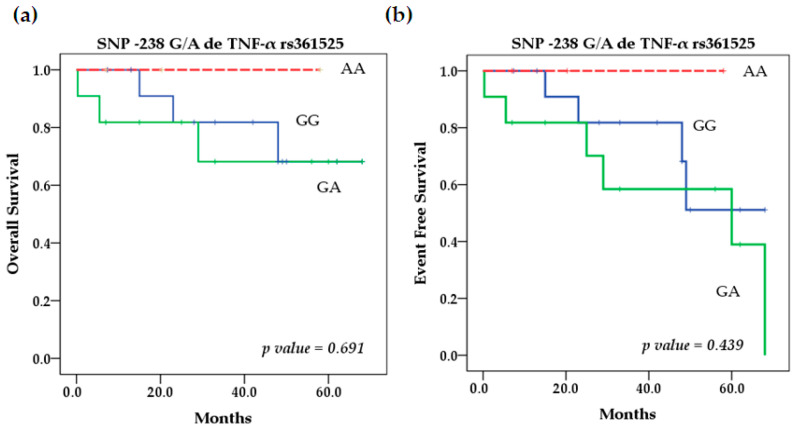

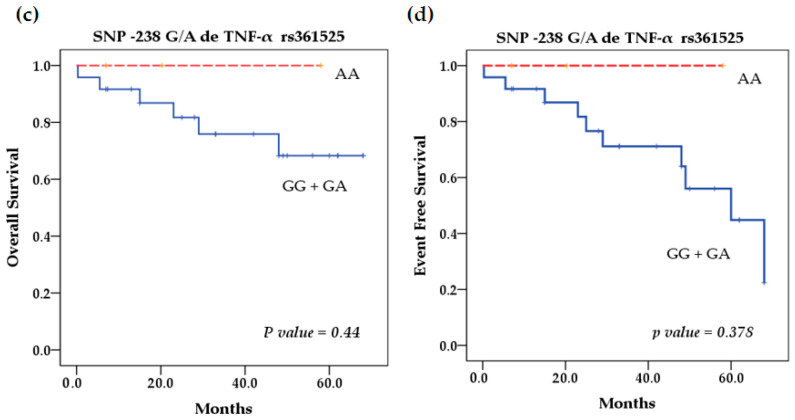
Kaplan–Meier curves of overall survival (**a**) and event-free survival (**b**) by genotype. Kaplan–Meier curves of overall survival (**c**) and event-free survival (**d**) stratifying GG + GA vs. AA genotype for SNP rs361525 TNF-α.

**Figure 2:**
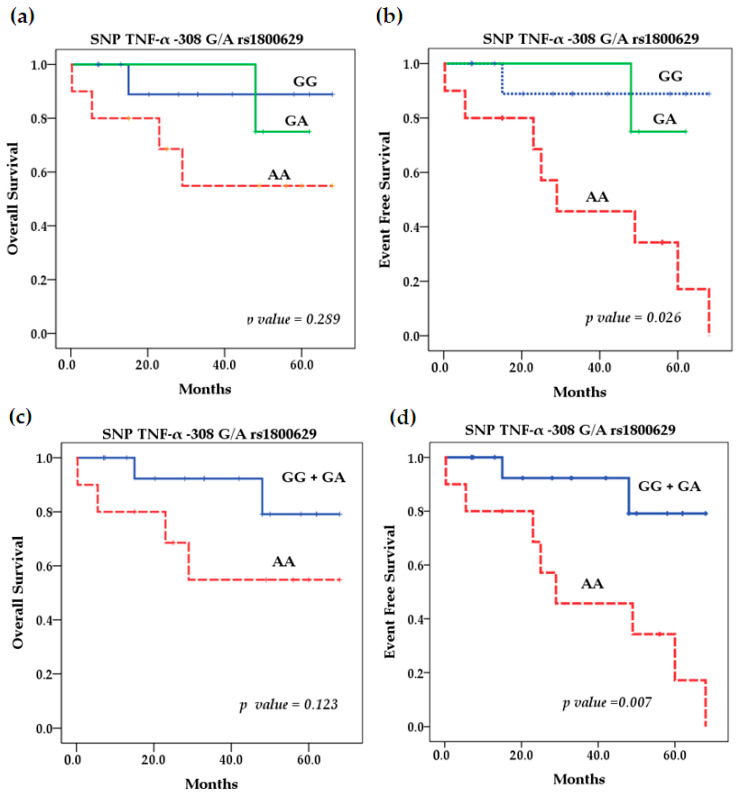
Kaplan–Meier curves of overall survival (**a**) and event-free survival (**b**) by genotype. Kaplan–Meier curves of overall survival (**c**) and event-free survival (**d**) stratifying GG + GA vs. AA genotype for SNP rs1800629 TNF-α.

**Figure 3:**
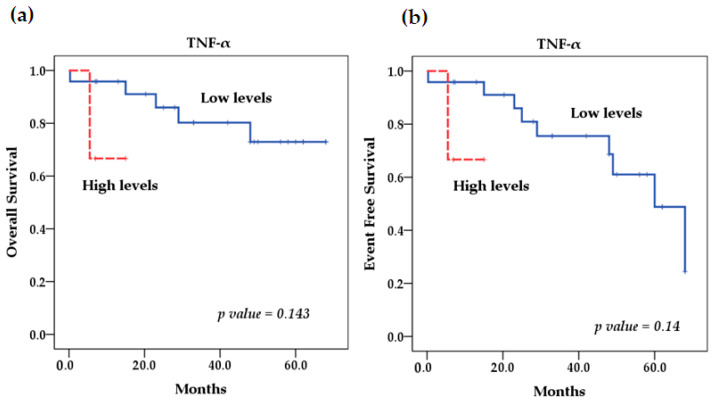
Kaplan–Meier curves comparing patients with high levels against those with low levels of TNF-α: (**a**) Overall survival (OS) and (**b**) event-free survival (EFS).

Our survival analysis using the Kaplan–Meier method showed that for the rs361525 TNF-α polymorphism, patients with NB and the AA genotype had better overall (OS) and event-free survival (EFS) rates. It was not significant (Figure 1). However, the TNF-α SNP rs1800629 had a significant effect (*p* = 0.026) on EFS because patients with the homozygous-variant AA genotype had lower EFS compared with patients with homozygous wild-type GG (92.35% vs. 20%) and heterozygous GA (75% vs. 20%) (Figure 2). On the other hand, OS and EFS in relation to the stratification of patients with high and low TNF-α serum levels did not show significant differences either (*p* = 0.14 and *p* = 0.143) (Figure 3). In addition, we performed multivariate Cox regression analysis, which was not significant; further details on the variables included and the results obtained can be found in Table A3.

We performed multivariate Cox regression analysis, which included the variables of age at diagnosis, *MYCN* status, INSS stage, and TNF-α serum concentrations and genetic variants rs1800629 and rs361525. It was not possible to adjust for *MYCN* amplification due to the reduced sample size. Our analyses showed no significant effects for these variants in conjunction with the risk of death Table A3

## 3. Discussion

TNF-α is a mediator of the immune response in various immunological diseases, infections, and types of cancer. The -238G/A and -308G/A polymorphisms are in the promoter region of the *TNF-α* gene and are associated with alterations in the levels of TNF-α expression, which may be related to the clinical outcomes of various diseases. In our study, we investigated the association of the genetic polymorphisms -238G/A rs361525 and 308 G/A rs1800629 and TNF-α serum concentrations with clinical outcomes in patients with NB. Previous studies analyzed the relationship of these genetic variants with various diseases and types of cancer, presenting contradictory results that largely depended on the ethnicity of the population and the type of disease [40,41,42]. Until now, there were no published studies about the role of *TNF-α* polymorphisms in NB.

In our study, for the -238 G/A rs361525 polymorphism, we found a significant difference in the frequency of the homozygous GG genotype (81.6% vs. 48.1%) in the control group compared with patients with NB. We observed the G allele an association with decrease in the risk of NB in the analyzed population. Likewise, we found an association between the GA heterozygous genotype of this variant and the risk of presenting NB. Our findings are consistent with previous reports in various meta-analysis studies that associated the -238 G/A polymorphism with the risk of various types of cancer, specifically the GA and AA genotypes with the risk of colorectal cancer in the Caucasian population [43,44,45,46]. The A allele and the GA and AA genotypes were associated with the risk of gastric cancer in Asian populations [41,42,43,44], the AA genotype was associated with the risk of hepatocellular carcinoma in African, Asian, and South American populations [45], and patients with genotypes GA + AA presented a higher risk of multiple myeloma in the Polish population [40]. Also, an association between the GA genotype of this polymorphism and cancer susceptibility in Polish patients with systemic sclerosis was recently reported [47]. However, multiple myeloma in the Turkish population has no association between this polymorphism and risk of disease [48], which is why ethnicity is an important factor that influences the incidences of genetic variants and their relationships with different diseases. Furthermore, our results contrast with those of other studies where the A allele of this polymorphism was presented as a biomarker of protection against diseases, such as lung cancer and idiopathic arthritis [29,30], as well as with reports where no association of SNP -238 G/A with the risk of diseases such as osteosarcoma, ischemic heart disease, and acute pancreatitis, among others [34,42,43,44,48]. In addition, results from studies on the Mexican population found no relationship between this polymorphism and the risk or severity of colorectal cancer [45].

The -308 G/A rs1800629 polymorphism regulates the expression of TNF-α, which may be associated with clinical outcomes in patients with NB. However, previous studies on this genetic polymorphism have produced contradictory results regarding the genotypes associated with the prognosis or development of various diseases. The results of several studies found no correlations between SNP -308 G/A and colorectal cancer, head and neck cancer, idiopathic arthritis, and acute pancreatitis, among others [30,42,44,45,46,48,49,50]. Contrary to these reports, in our study we found a significant difference in the frequency of the homozygous GG genotype (81.6% vs. 48.1%) in the control group compared with patients with NB. In addition, the G allele in its homozygous (GG) or heterozygous (GA) forms showed a decreased risk of NB, which indicates that this allele may be a probable biomarker of protection in this disease. On the contrary, we found an association between the AA genotype and the risk of NB, and for carriers of the A allele, it may be a probable biomarker of risk for this disease. This is consistent with a study showing that carriers of at least one A allele of this polymorphism are significantly associated with a two-fold increased risk of developing relapsing-remitting multiple sclerosis [51]. We also observed the GA/AA genotypes showed considerable associations with poor prognostic factors, such as high risk, INSS stage 4, and unfavorable histology. Likewise, we found the variant AA homozygous genotype to be considerably associated with lower EFS in patients with NB. Some of our findings are consistent with reports from other research groups, such as in studies of breast cancer, which also reported the GA and AA genotypes of the -308 G>A polymorphism contributed considerably to the risk of this disease in the Mexican population [34]; likewise, the GA genotype is associated with lower overall survival and EFS in Lithuanian women with breast cancer [49]. Similarly, in several reports and meta-analysis studies, the AA or GA genotypes of this variant are associated with the risk of various malignant diseases, such as osteosarcoma, gastric or colorectal cancer, and hepatocellular carcinoma, and the A allele is also associated with the development and progression of lung cancer, as well as the development of metastasis in rectal cancer [29,34,44,45]. However, there is a report that associates the homozygous wild-type GG genotype with the risk of multiple myeloma compared with a group of healthy controls [41]. These reported differences may be due to the sample size, as well as the genetic differences of the populations studied. The Mexican population is ethnically heterogeneous, and the results of reports on the distribution of cytokine polymorphisms, such as TNF-α, are different in the Mexican population compared with other ethnic groups, including Caucasians, Asians, and Africans [32]. This is why knowledge of the distribution of these polymorphisms in the Mexican population may be relevant in understanding their participation as genetic markers of susceptibility in Mexico.

In our study in the analysis of the polymorphisms evaluated with the clinicopathologic factors of the patients, we did not obtain results related to *MYCN* status, due to the low frequency of occurrence of this oncogene. On the other hand, we observed that most patients carrying the G allele for SNP-238 G/A rs361525 presented tumors in disseminated stages (INSS3 and INSS4) (*p* = 0.017) (Table 3); the G allele of this polymorphism is associated with elevated TNF-α production compared with the A allele [28]. Therefore, we suppose that this cytokine could probably be involved in the progression of this type of tumors; however, reports are limited and do not yet provide clear evidence of the role of this SNP in the regulation of *TNF-α* gene transcription. On the other hand, analysis of the SNP -308 G/A rs1800629 *TNF-α* SNP with clinicopathologic factors of patients with NB, we only observed that the majority of patients with A allele presented high-risk NB (*p* = 0.04) and unfavorable tumor histology (*p* = 0.03) (Table 4); it has been reported that the presence of the -308A allele correlates with increased TNF-α production in vitro and in vivo [21,22]. These associations related to tumor histology and staging based on the INSS staging system are important because NB treatment should be performed based on risk groups; among the most important clinical factors are age, tumor location and histology, and stage based on INSS criteria. However, these findings should be taken with caution because, from a statistical point of view, it would be important to increase the power of the tests to obtain associations with better significance values, so it is necessary to increase the sample size.

The clear distinction that we observed between the levels of TNF-α in patients with NB versus the control group may indicate that high serum levels of this cytokine may have an implication in the development of NB. This can be explained by the fact that TNF-α is a potent immunostimulatory cytokine with local effects in the tumor microenvironment and possible systemic effects; it can also contribute to the maintenance of a pro-inflammatory environment [11]. However, there are still discrepancies in this regard because it has been reported that TNF-α acts as an autocrine growth factor for NB cells; on the other hand, an in vitro study reported that TNF-α inhibited NB cell growth and DNA synthesis [52]. In addition, the increased TNF-α serum levels in cancer patients are associated with adverse disease outcomes [50]. We found a significant difference (*p* < 0.001) in TNF-α serum levels between patients with neuroblastoma and the control group. Other investigations produced similar results in other diseases such as lung and colorectal cancer and osteosarcoma, among others [53,54,55]. However, when stratifying the cytokine’s serum concentrations into low and high levels, considering the previously established cut-off values, we observed no considerable differences between the group with NB and the healthy individuals. It is important to mention that the population considered for the estimation of the cut-off values was between the ages of 0 and 17 years, which is the criterion for the pediatric population in our country. On the other hand, the control group used in this work was matched by age with the patients with NB, so the age range was from 0 to 8 years; for this reason, we consider that the differences between the control group and the cases with NB are important because they are within the same age group and the fact that when performing the stratification at low and high levels considering the cut-off values, this difference was not significant, may be because the group to obtain the cut-off values included children with a higher age range than the patients included in this study. This is related to what was previously reported by authors who indicate that age clearly influences the cytokine expression profiles in healthy children [56,57], reporting that several cytokines, including TNF-α, show an increase associated with age, suggesting that cytokine expression dynamics in infancy should be taken into account when evaluating them in diagnostic assays or as immunological biomarkers [57].

TNF-α expression is mainly regulated at the transcriptional level and some polymorphisms in the gene that codes for this protein are related to the production of TNF-α. We found no significant associations between the genotypes of polymorphisms -238 G/A rs361525 and -308 G/A rs1800629 and low and high TNF-α levels in patients with NB. Our results agree with those of other studies that also found no relationship between TNF-α serum concentrations and these SNPs [45]. However, our results contrast with several trials that found a significant relationship between elevated TNF-α serum levels and patients carrying the -308 A allele of the rs1800629 variant [38,58,59,60]; in the case of SNP rs361525, the -238A allele is associated with decreased TNF-α serum levels [20,21,60]. Probably, in our study, we did not observe a relationship between polymorphisms analyzed and serum concentrations of TNF-α due to the size of the sample included. It would also be productive to analyze other genetic variants of the *TNF-α* promoter gene that could affect transcription, such as SNP -863 C/A, which has been associated with reduced levels of TNF-α [61]. On the other hand, high TNF-α expression has been described as a predictor of poor survival in cancer patients [62,63]. In our case, the levels of this cytokine showed no association with survival in the studied population. Our results may be due to the small sample size. In addition, we only performed a measurement of the TNF-α concentration in serum at the time of diagnosis; however, it is important to evaluate the serum levels at various stages of the disease evolution to examine whether the increase in cytokine circulation can affect the development and prognosis of NB.

It is also important to consider larger cohort studies in the future, as well as additional prospective and retrospective studies. In addition, it is necessary to evaluate the serum levels of TNF-α during the disease and to analyze the expression of the TNF-α receptors (TNFR-1 and TNFR-2). These determinations, together with the genotyping of genetic polymorphisms, could contribute to improving our understanding of the role of TNF-α in NB.

## 4. Materials and Methods

In this study, we included a total of 27 pediatric patients diagnosed with neuroblastoma from the Hospital Infantil de México Federico Gómez from December 2015 to December 2019. The staging of Neuroblastoma tumors in this study was performed based on clinical, radiographic, and surgical evaluation, according to the criteria of the International Neuroblastoma Staging System (INSS) and the International Neuroblastoma Risk Group (INRG) [64,65]. The degree of differentiation of neuroblastic tumors was determined based on histopathological criteria following the classification parameters of the International Neuroblastoma Pathology Classification (INCP), for which histopathological and immunohistochemical studies were performed on the tumor material, such as the detection of the NB84 marker, morphometric evaluation to study ploidy, and molecular analysis to determine MYCN amplification [66,67]. The histology according to the Shimada system, was based on the amount of Schwannian stroma, the degree of differentiation, the mitosis–karyorrhexis index (MKI), and age at diagnosis, classifying the tumors into two groups of favorable and unfavorable histological prognosis [9,64]. We collected paraffin-embedded tissue samples and peripheral blood at the time of diagnosis. We obtained the characteristics of each patient, such as age at diagnosis, risk, stage, and site of the primary tumor, histology, clinical follow-up time, and prognostic factors, from clinical records. The control group was age- and sex-matched with NB cases; 38 healthy children were included, without presenting any infectious or inflammatory processes or immune diseases at the time of sample collection.

### 4.1. Ethical Considerations

Our research was evaluated and approved by the Ethics Committee of the Institute (number CONBIOÉTICA-09-CEI-010-20160627). Informed consent was provided by the legal guardians of all patients and healthy controls. We carried out this project in accordance with the principles established by the Helsinki World Medical Assembly and all the applicable modifications established by the World Medical Assembly and the ICH guidelines for Good Clinical Practice (GCP), as well as the regulations of the General Law of Health in Research for the Health of Mexico.

### 4.2. Determination of MYCN Gene Amplification

To detect *MYCN* gene amplification, we obtained DNA from formalin-fixed, paraffin-embedded samples of NB using the QIAamp DNA FFPE Tissue kit (Qiagen, Hilden, Germany) according to the manufacturer’s instructions. For healthy controls, DNA was obtained from peripheral blood samples with the DNA Blood Midi kit (Qiagen, Hilden, Germany) according to the manufacturer’s instructions. We analyzed the *MYCN* copy number by real-time PCR and used the commercial TaqMan copy number assay for the *MYCN* gene (ID Hs00658058) from Thermo Fisher Scientific Applied Biosystems (Thermo Fisher Scientific, Foster, CA, USA) according to the manufacturer’s instructions.

### 4.3. Analysis of TNF-α Genetic Polymorphisms

**Figure A1:**
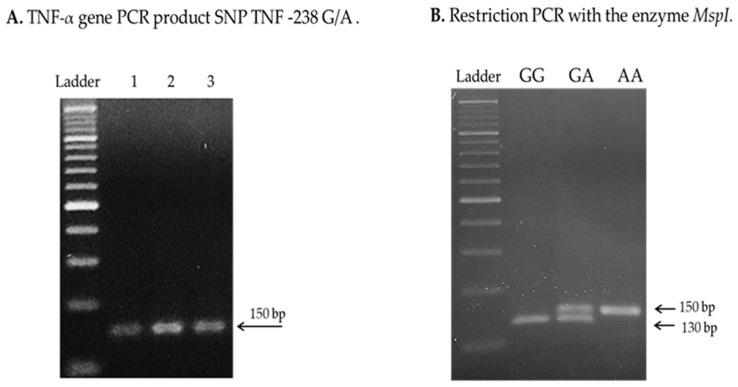
Polymorphism analysis of the *TNF-α* -238 G/A by PCR-RFLP. (**A**) shows the 100 bp molecular weight marker and three shows the 150 bp PCR product. (**B**) shows the enzymatic digestion of the PCR product with the *MspI* enzyme, lane 1 was a 100 bp molecular weight marker, lanes 2 to 4 show representative samples of the GG, GA, and AA genotypes for this SNP.

**Figure A2:**
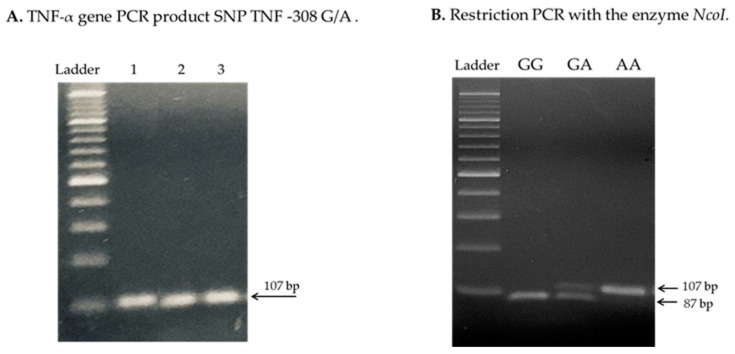
Polymorphism analysis of the *TNF-α* -308 G/A by PCR-RFLP. (**A**) shows the 100 bp molecular weight marker and three shows the 107 bp PCR product. (**B**) shows the enzymatic digestion of the PCR product with the *NcoI* enzyme, lane 1 was a 100 bp molecular weight marker, lanes 2 to 4 show representative samples of the GG, GA, and AA genotypes for this SNP.

We obtained peripheral blood samples (2 mL) in EDTA tubes to obtain genomic DNA. We extracted blood with the DNA Blood Midi Kit (Qiagen, Hilden, Germany) according to the manufacturer’s instructions. We used the PCR-RFLP (polymerase chain reaction–restriction fragment length polymorphisms) method to determine the *TNF-α* genetic polymorphisms. The PCR mixture contained: 0.1 µg DNA, 2.5 mM dNTPs, 3 mM MgCl_2_, 1X buffer (20 mM Tris-HCl and 2 mM MgSO_4_), 10 pm oligonucleotides, and 0.5 U DNA Taq polymerase (New England Biolabs, Beverley, MA, USA). For restriction fragment length polymorphism analysis, the PCR products were purified before restriction using the MinElute PCR Purification Kit (Qiagen, Hilden, Germany). We digested the PCR products with specific restriction enzymes. We analyzed the -238 G>A polymorphism using the forward and reverse primers 5′-ATCTGGAGGAAGCGGTAGTG-3′ and 5′-AGAAGACCCCCCTCGGAACC-3′. We used a PCR protocol of 94 °C for 3 min, 35 cycles of 94 °C for 30 s, 57 °C for 30 s, 72 °C for 30 s, and a final extension at 72 °C for 5 min, and we cut 10 µL of the 150 bp PCR product using 2U of the *MspI* restriction enzyme (New England Biolabs, Beverley, MA, USA) at 37 °C for 4 h. The restriction fragments for the genotypes included GG: 130 pb and 20 pb; GA: 150, 130, and 20 pb; and AA: 150 pb Figure A1. For the -308 G>A polymorphism, we used the forward and reverse primers 5′-AGGCAATAGGTTTTGAGGGCCAT-3′ and 5′-TCCTCCCTGCTCCGATTCCG-3′. We used a PCR protocol of 94 °C for 3 min, 35 cycles of 94 °C for 30 s, 61 °C for 30 s, 72 °C for 30 s, and a final extension at 72 °C for 5 min, and we cut the 107 bp PCR product using 2 U of the *NcoI* restriction enzyme (New England Biolabs, Beverley, MA, USA) at 37 °C for 4 h. The restriction fragments for the genotypes included GG: 87 bp and 20 bp; GA: 107, 87, and 20 bp; and AA: 107 bp Figure A2. We analyzed the PCR and enzyme restriction products by electrophoresis in 2.5% agarose gels stained with ethidium bromide, and we visualized them with UV light to detect each genotype.

### 4.4. Measurement of TNF-α Serum Levels

Of the 38 healthy children, we selected 27 to evaluate serum cytokine levels; these were age- and gender-matched with Neuroblastoma patients to avoid any bias because serum cytokine levels may vary by age and gender. We obtained the blood samples in tubes with separating gel for serum analysis. The blood remained for 1 h to complete the clot retraction. Later, we centrifuged for 15 min at 3500 rpm. We stored serum at −70 °C in 500 µL aliquots and subsequently analyzed the immunological markers. We quantified TNF-α in the serum of NB patients and controls by the ELISA method using the BD OptEIA ELISA Kit (BD Biosciences, Franklin Lakes, NJ, USA) according to the manufacturer’s instructions. We analyzed the samples in duplicate, expressed the concentration in pg/mL, calculated by interpolation to a TNF-α standard curve, and considered the dilution factor 1:5. To establish cut-off lines for TNF-α, we previously performed a preliminary analysis that included 30 serum samples from the pediatric population from 0 to 17 years in which inflammatory history and chronic degenerative disorders were ruled out, and 3 negative controls to obtain the value of the mean of the determinations ± 2 standard deviations (SD), to establish the cut-off values of the serum levels of TNF-alpha in the pediatric population. TNF-alpha concentrations were quantified from the logistic curve of the TNF-α standard; the specificity of the test is 98%, according to the manufacturer’s instructions. The cut-off point of TNF-α obtained was 21 pg/mL; in this way, the values for the stratification of serum levels of TNF-α were obtained, considering low levels ≤ 20 pg/mL and high levels ≥ 21 pg/mL, which coincide with what was reported by Noori et al. 2017 [68].

### 4.5. Statistical Analysis

We performed the statistical and graphical analyses of this study using STATA version 13 and SPSS version 19 programs. Descriptive data were represented in terms of frequencies. Differences between clinical data categorical variables of patients and genotypes were performed using Fisher’s exact test. To assess the association between genetic polymorphisms with the presence of the disease and prognostic factors, we estimated the odds ratios (OR) by simple logistic regression analysis. The Hardy–Weinberg genetic equilibrium (EH-W) was determined using the χ2 test. The comparison of serum concentrations of TNF-α between the group of patients with NB and the control group was performed using the Wilcoxon test. Survival analysis was performed using the Kaplan–Meier method, and the differences between groups were estimated by the log rank test; for overall survival (OS), it was calculated from the date of diagnosis to the date of death or last contact with patient, and event-free survival (EFS) was considered from the date of diagnosis to the date of relapse or death. The Cox model was used to identify prognostic variables that affected overall survival. The level of statistical significance considered for all tests was a value of *p* < 0.05.

## 5. Conclusions

We identified a possible association between the *TNF-α* genetic polymorphisms -238 G/A rs361525 and -308 G/A rs1800629 and the risk of neuroblastoma. Patients with NB carrying the GG genotype for SNP rs1800629 had a better prognosis than those with the AA genotype, who had low EFS, for which the A allele may play an important role in the progression of NB as probable factors associated with, and useful for, identifying high-risk patients and following up the disease in Mexican patients with neuroblastoma. Circulating TNF-α serum concentrations were different between patients with NB and healthy controls; however, we found no relationship between the analyzed TNF-α serum levels and SNP genotypes. It should be noted that this is a preliminary study, and it is important to conduct research on a larger scale, through inter-institutional studies, to further evaluate the contribution of *TNF-α* genetic polymorphisms as potential markers of treatment response, disease progression, and severity.

## Figures and Tables

**Table 1:** Characteristics and clinical features of the study population.

Characteristics		Patients n = 27 (%)	Healthy Controls n = 38 (%)
Age	<18 months	16 (59.3)	22 (57.9)
	18 months–5 years	6 (22.2)	9 (23.7)
	>5 years	5 (18.5)	7 (18.4)
Sex	Male	10 (37.03)	14 (36.85)
	Female	17 (62.97)	24 (63.15)
INSS stage	1	5 (18.5)	-----
	2a	0 (0)	-----
	2b	2 (7.5)	-----
	3	7 (25.9)	-----
	4	13 (48.1)	-----
	4S	0 (0)	-----
INRG	L1	5 (18.5)	-----
	L2	8 (29.6)	-----
	M	14 (51.9)	-----
	MS	0 (0)	-----
Risk	Low	5 (18.5)	-----
	Intermediate	7 (25.9)	-----
	High	15 (55.6)	-----
Primary Tumor Site	Adrenal	8 (29.6)	-----
	Retroperitoneal	11 (40.7)	-----
	Paraspinal	3 (11.1)	-----
	Abdomen/Pelvic	1 (3.7)	-----
	Mediastinal	4 (14.9)	
Differentiation	Undifferentiated	2 (7.4)	-----
	Partially differentiated	16 (59.3)	-----
	Differentiated	5 (18.5)	-----
	Not specified	4 (14.8)	-----
Histology (Shimada)	Favorable	15 (55.6)	-----
	Unfavorable	12 (44.4)	-----
*MYCN*	Amplified	1 (3.7)	-----
	Not amplified	26 (96.3)	38 (100)
Relapse	Yes	7 (25.9)	
Death	Yes	6 (22.2)	

INSS = International Neuroblastoma Staging System, INRG = International Neuroblastoma Risk Group, *MYCN* = *MYCN* proto-oncogene.

**Table 2:** Distribution of genotypes and alleles of polymorphisms -238 G/A rs361525 and -308 G/A rs1800629 of *TNF-α* in NB patients and control group.

SNP	Controls (%) n = 38	NB Patients (%) n = 27	OR (95% CI)	*p* Value
*TNF-α* -238 G/A rs361525				
GG	31 (81.6)	13 (48.1)	Ref	
GA	7 (18.4)	11 (40.7)	4.52 (1.61–12.67)	0.004
AA	0 (0)	3 (11.1)	-----	---
GA + AA vs. GG	---	---	0.20 (0.068–0.63)	0.006
Allele G	69 (90.79)	37 (68.5)	----	----
Allele A	7 (9.21)	17 (31.5)	----	----
*TNF-α* -308 G/A rs1800629				
GG	33 (86.8)	13(48.2)	Ref	
GA	1 (2.6)	4 (14.8)	-----	
AA	4 (10.5)	10 (37)	2.89 (1.452–5.769)	0.003
GG + GA vs. AA	----	----	0.10 (0.030–0.393)	0.001
GA + AA vs. GG	----	----	5 (1.36–18.3)	0.015
Allele G	67 (88.15)	30 (55.6)	---	--
Allele A	9 (11.84)	24 (44.4)	----	----

SNP: Single nucleotide polymorphisms, NB: Neuroblastoma, OR: Odds ratio, CI: Confidence interval, ref.: reference genotype, vs.: versus, HWE: Hardy–Weinberg equilibrium. HWE rs361525 controls (X^2^ = 0.39, *p* = 0.53), cases (X^2^ = 0.08, *p* = 0.77), HWE rs1800629 controls (X^2^ = 29.02, *p* < 0.05) and cases (X^2^ = 13.2, *p* < 0.05).

**Table 3:** The association between rs361525 *TNF-α* polymorphism stratified by genotype GG + GA versus AA and the clinicopathologic factors of NB patients.

Variables	GG + GA	AA	OR * (95% CI)	*p* Value
Risk				
Low	3 (11.1)	2 (7.4)	1.5 (0.20–10.8)	0.05
Intermediate/High	21 (77.7)	1 (3.7)		
INSS stage				
stage 1, 2	5 (18.5)	3 (11.1)	0.043 (0.003–0.57)	0.017
stage 3, 4	19 (70.3)	1 (3.7)		
Histology (Shimada)				
Favorable	13 (48.1)	2 (7.4)	1.69 (0.134–21.26)	0.68
Unfavorable	11 (40.7)	1 (3.7)		
*MYCN*				
Amplified	1 (3.7)	----	NC	NC
Not amplified	23 (85.2)	3 (11.1)		
Death				
Yes	6 (22.2)	--	NC	NC
No	18 (66.7)	3 (11.1)		

OR * = unadjusted odds ratio; CI = confidence interval; NC = not calculable; INSS = International Neuroblastoma Staging System; *MYCN* = *MYCN* proto-oncogene.

**Table 4:** The association between rs1800629 *TNF-α* polymorphism stratified by genotype GG versus GA + AA and the clinicopathologic factors of NB patients.

Variables	GG	GA + AA	OR * (95% CI)	*p* Value
Risk				
Low/Intermediate	9 (33.3)	3 (11.1)	2.07 (0.39–10.8)	0.04
High	4 (14.8)	11 (40.7)		
INSS stage				
stage 1, 2	6 (22.2)	1 (3.7)	0.71 (0.07–6.53)	0.76
stage 3,4	7 (25.9)	13 (48.1)		
Histology (Shimada)				
Favorable	10 (37)	5 (18.5)	6 (1.10–32.5)	0.03
Unfavorable	3 (11.1)	9 (33.3)		
*MYCN*				
Amplified	---	1 (3.7)	NC	NC
Not amplified	13 (48.1)	13 (48.1)		
Death				
Yes	1 (3.7)	5 (18.5)	0.15 (0.01–1.51)	0.10
No	12 (44.4)	9 (33.3)		

OR * = unadjusted odds ratio; CI = confidence interval; NC = not calculable; INSS = International Neuroblastoma Staging System; *MYCN* = *MYCN* proto-oncogene.

**Table 5:** Analysis of serum concentrations of TNF-α between NB cases and the control group.

TNF-α (pg/mL)	Controls n = 27	NB Cases n = 27
Range	1.8–3	7.8–80.1
Mean	2.474	11.851
Standard deviation (SD)	0.358	14.86
*p* value	<0.001	
Low levels of TNF-α (≤20 pg/mL)	27 (100)	24 (88.9)
High levels of TNF-α (≥21 pg/mL)	------	3 (11.1)

## Data Availability

The data presented in this study are available on request from the corresponding author. The data are not publicly available due to confidentiality of patient data.

## Appendix A

**Table A1:** Genotype frequencies of -238 G/A rs361525 and -308 G/A rs1800629 *TNF-α* and prognostic factors of NB patients.

	SNP -238 G/A rs361525 n = 27 (%)		SNP -308 G/A rs1800629 n = 27 (%)	
Variables	GG + GA	AA	GG	GA + AA
INSS stage				
1	3 (11.1)	2 (7.4)	5 (18.5)	----
2b	2 (7.4)	----	1 (3.7)	1 (3.7)
3	7 (25.9)	----	3 (11.1)	4 (14.8)
4	12 (44.4)	1 (3.7)	4 (14.8)	9 (33.3)
*p* value	0.218		0.05	
INRG				
L1	3 (11.1)	2 (7.4)	3 (11.1)	2 (7.4)
L2	8 (29.6)	----	5 (18.5)	3 (11.1)
M	13 (48.1)	1 (3.7)	5 (18.5)	9 (33.3)
MS	----	---	---	
*p* value	0.146		0.51	
Risk				
Low	3 (11.1)	2 (7.4)	4 (14.8)	1 (3.7)
Intermediate	7 (25.9)	----	5 (18.5)	2 (7.4)
High	14 (51.9)	1 (3.7)	4 (14.8)	11 (40.7)
*p* value	0.126		0.05	
Differentiation				
Undifferentiated	2 (7.4)	----	----	2 (7.4)
Partially differentiated	14 (51.9)	2 (7.4)	9 (33.3)	7 (25.9)
Differentiated	4 (14.8)	1 (3.7)	3 (11.1)	2 (7.4)
Not specified	4 (14.8)	----	1 (3.7)	3 (11.1)
*p* value	1		0.414	
Histology (Shimada)				
Favorable	13 (48.1)	2 (7.4)	10 (37)	5 (18.5)
Unfavorable	11 (40.7)	1 (3.7)	3 (11.1)	9 (33.3)
*p* value	0.586		0.038	
*MYCN*				
Amplified	1 (3.7)	-----	----	1 (3.7)
Not amplified	23 (85.2)	3 (11.1)	13 (48.1)	13 (48.1)
*p* value	0.296		0.3	

*p* value obtained by X analysis and Fisher exact test

**Table A2:** Association of polymorphism rs1800629 and rs361525 genotypes with serum levels of TNF-α in patients with NB.

	Low Serum TNF-α Levels	High Serum TNF-α Levels	Value *p*	OR (95% CI)
*TNF-α* -308 G/A rs1800629				
GG	12 (44.44)	1 (3.7)	Ref	----
GA	4 (14.8)	0	---	---
AA	8 (29.6)	2 (7.4)	0.145	2.63 (0.71–9.70)
GG + GA vs. AA	-----	-----	0.119	7.27 (0.60–87.84)
GA + AA vs. GG	-----	-----	0.214	0.20 (0.017–2.47)
*TNF-α* -238 G/A rs361525				
GG	13 (48.14)	0	Ref	----
GA	8 (29.6)	3 (11.1)	0.118	3.87 (0.71–21.12)
AA	3 (11.1)	0	-----	------
GG + GA vs. AA	-----	----	---	NC
GA + AA vs. GG	-----	----	----	NC

CI = Confidence interval; OR = odds ratio; ref = reference genotype; vs. = versus; NC = not calculable.

**Table A3:** Results of the multivariate Cox proportional hazards model.

Model	HR (95% CI)	*p* Value
A		
*TNF-α* rs361525 (GG + GA vs. AA)	NC	NC
Age (≥18 months vs. <18 months)	1.16 (0.16–8.39)	0.87
INSS stage (4 vs. 1, 2, 3)	5.62 (0.51–61.6)	0.15
*MYCN* (amplified vs. not amplified)	NC	NC
B		
*TNF-α* rs1800629 (GG + GA vs. AA)	2.08 (0.35–12.30)	0.41
Age (≥18 months vs. <18 months)	1.04 (0.14–7.64)	0.96
INSS stage (4 vs. 1, 2, 3)	5.16 (0.41–64.3)	0.20
*MYCN* (amplified vs. not amplified)	NC	NC
C		
Serum TNF-α levels (High vs. Low)	4.95 (0.30–79.5)	0.25
Age (≥18 months vs. <18 months)	1.74 (0.20–15.12)	0.61
INSS stage (4 vs. 1, 2, 3)	4.91 (0.48–49.35)	0.17
*MYCN* (amplified vs. not amplified)	NC	NC

HR = hazard ratio; CI = confidence interval; vs. = versus; NC = not calculable.
